# Differential Proteomic Analysis by iTRAQ Reveals the Mechanism of *Pyropia haitanensis* Responding to High Temperature Stress

**DOI:** 10.1038/srep44734

**Published:** 2017-03-17

**Authors:** Jianzhi Shi, Yuting Chen, Yan Xu, Dehua Ji, Changsheng Chen, Chaotian Xie

**Affiliations:** 1 Fisheries College, Jimei University, Xiamen 361021, P. R. of China; 2 Key Laboratory of Healthy Mariculture for the East China Sea, Ministry of Agriculture, Xiamen 361021, P. R. of China

## Abstract

Global warming increases sea temperature and leads to high temperature stress, which affects the yield and quality of *Pyropia haitanensis*. To understand the molecular mechanisms underlying high temperature stress in a high temperature tolerance strain Z-61, the iTRAQ technique was employed to reveal the global proteomic response of Z-61 under different durations of high temperature stress. We identified 151 differentially expressed proteins and classified them into 11 functional categories. The 4 major categories of these are protein synthesis and degradation, photosynthesis, defense response, and energy and carbohydrate metabolism. These findings indicated that photosynthesis, protein synthesis, and secondary metabolism are inhibited by heat to limit damage to a repairable level. As time progresses, misfolded proteins and ROS accumulate and lead to the up-regulation of molecular chaperones, proteases, and antioxidant systems. Furthermore, to cope with cells injured by heat, PCD works to remove them. Additionally, sulfur assimilation and cytoskeletons play essential roles in maintaining cellular and redox homeostasis. These processes are based on signal transduction in the phosphoinositide pathway and multiple ways to supply energy. Conclusively, Z-61 establishes a new steady-state balance of metabolic processes and survives under higher temperature stress.

*Pyropia* is an economically important marine red alga used for food, fertilizer, medicine, and chemicals. The farming and processing of *Pyropia* has become one of the largest seaweed industries in East Asian countries, including China, Japan, and South Korea^1^,^2^,^3^. *Pyropia haitanensis*, a typical warm temperate zone species originally found south of China, has been extensively cultured in the Fujian, Zhejiang, and Guangdong Provinces for more than 50 years, and its output accounts for about 75% of the total production of *Pyropia* in China^2^.

Temperature is the major environmental factor that affects the development, metabolism, productivity, and quality of *P. haitanensis*. Under natural conditions, *P. haitanensis* is cultured from September to March of the next year, and the optimal seawater temperature is 14 °C to 26 °C. However, in the early seeding period (September to October in south China), *P. haitanensis* farms often suffer from consistently high temperatures or the well-known “temperature rebound” as temperature increases following a dramatic drop to temperatures suitable for conchospore release and germination^4^. The persistence of high temperatures can negatively affect *P. haitanensis* blades by inhibiting the survival and division of these conchospores. In addition, germlings become prone to disease, premature senility, and eventual decay, leading to a substantial reduction in yield^5^. In recent years, high temperatures have markedly affected *P. haitanensis* cultivation and reduced its yield^6^. Furthermore, according to reports from the Intergovernmental Panel on Climate Change, high temperature stresses will increase in the near future due to global climate change. To adapt to global climate change and sustain *Pyropia* cultivation, several high temperature tolerance strains (Z-26, Z-61, and ZS-1) of *P. haitanensis* have been selected and widely cultivated in South China^5^,^7^. Therefore, a better understanding of the high temperature tolerance mechanisms in these tolerant strains would lay the theoretical foundation for further cultivar breeding of *Pyropia*.

The mechanisms of responding to high temperature stress have been investigated in a great deal of higher plants, especially in model plants and economic crops, and various mechanisms have been summarized. These include membrane stability maintenance, Reactive oxygen species (ROS) scavenging, antioxidants production, the accumulation and adjustment of compatible solutes, the induction of mitogen-activated protein kinase (MAPK) and calcium-dependent protein kinase (CDPK) cascades, and, most importantly, chaperone signaling and transcriptional activation^8^,^9^,^10^,^11^. However, much less is known about the mechanisms of high temperature tolerance in algae. To understand the physiology and molecular mechanisms underlying *P. haitanensis* high temperature stress responses, Zhang *et al*. compared the physiological response of a high temperature tolerance strain (Z-61) to that of a wild-type strain, and found that Z-61 induces its antioxidant and osmoregulation systems, whereas the wild-type strain only induces its osmoregulation system during high temperature stress^3^. Furthermore, the baseline activity of the antioxidant enzymes differed significantly between the 2 strains^3^. Recently, high throughput experimental methods such as transcriptomics and proteomics were also utilized to identify stress-responsive genes and proteins regulated by elevated temperatures in *Pyropia*. Transcriptomic work in Z-61 revealed that its high temperature response is characterized by rapid reprogramming of gene expression machinery leading to up- and down-regulation of thousands of genes within minutes of exposure to high temperature stress^12^. Additionally, the stress-related genes involved in several pathways have been identified, such as redox homeostasis, photosynthesis, energy metabolism, polysaccharide and cell wall metabolism, and defense. However, since changes in transcription often do not correspond with changes in protein expression^13^, comparative proteomic analysis based on 2-dimensional electrophoresis (2-DE) was conducted to screen the differentially expressed proteins in Z-61 under normal temperature and high temperature stress. Fifty-nine differentially expressed proteins, which involved processes such as photosynthesis, energy metabolism, redox homeostasis, response to stimuli, were identified^6^.

Proteins represent biochemical machinery and the functional processes of how an organism responds to a changing environment. Meanwhile, proteomics presents an effective approach to pursue a systems-based perspective of how proteins change, and thus how organisms adapt to various abiotic environments^14^. However, traditional gel-based proteomics is biased towards hydrophilic proteins with higher abundances and intermediate Mr and pI properties. Therefore, this technique is not sufficient for higher coverage and more accurate quantification in proteomic studies^15^. In recent years, new mass spectrometry-based proteomic techniques, such as isobaric tags for relative and absolute quantitation (iTRAQ) and Isotope-coded affinity tags (ICAT), have been applied for proteomic analysis. Among them, iTRAQ is the most popular technique in plant quantitative proteomics^16^,^17^. This technique is based on tagging the N-terminus of peptides generated from tryptic protein digests, which overcomes some limitations of gel-based techniques and also improves the throughput of proteomic studies. Furthermore, this technique has a high degree of sensitivity, and the amine specific isobaric reagents permit the identification and quantitation of up to 8 different samples simultaneously^16^,^17^. Thus, in this paper, we employed iTRAQ and liquid chromatography-tandem mass spectrometry (LC-MS/MS) to further understand the molecular mechanisms underlying high temperature stress in *Pyropia* and reveal the global proteomic response of the Z-61 stains of *P. haitanensis* under high temperature stress.

## Results

### Overview of Quantitative Proteomics

A total of 269614 spectra were obtained from the iTRAQ-LC-MS/MS proteomic analysis of all Z-61 samples. After data filtering to eliminate low-scoring spectra, a total of 97072 unique spectra that met the strict confidence criteria for identification were matched to 1895 unique proteins (Supplementary Information Tables S1 and S2).

**Figure 1:**
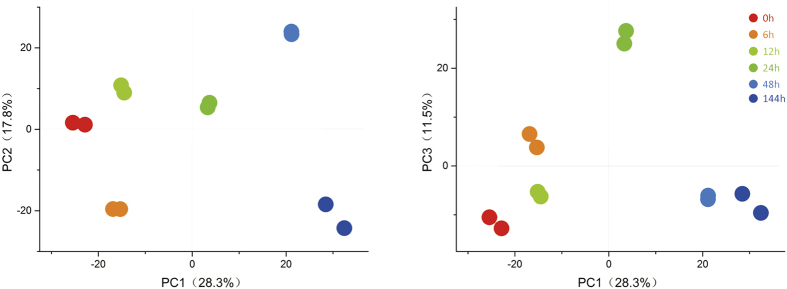
PCA of iTRAQ data in each treatment of high temperature stress. Numbers in parentheses represent the percentage of total variance explained by the first, second, and third PC. The 2 biological replicates of a treatment are represented by dots of the same color.

To assess the reproducibility of the iTRAQ data, PCA was performed on every 2 replicates at each time point (Fig. 1). Results show that the iTRAQ data in 2 replicates at each time point were almost unanimous, and different time points were clearly separated, suggesting that protein abundance changed with prolonged high temperature stress.

### Expression Profile of Differentially Expressed Proteins

**Figure 2:**
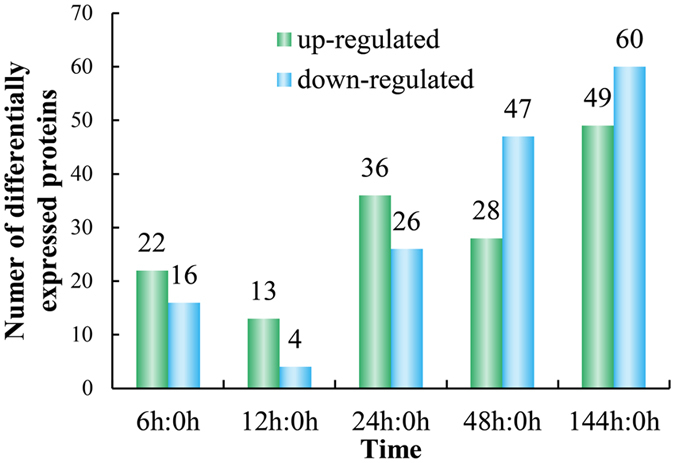
Numbers of differentially expressed proteins in each comparison. The numbers above the bars show the quantity of up-(green) and down-(blue) regulated expression proteins.

**Figure 3:**
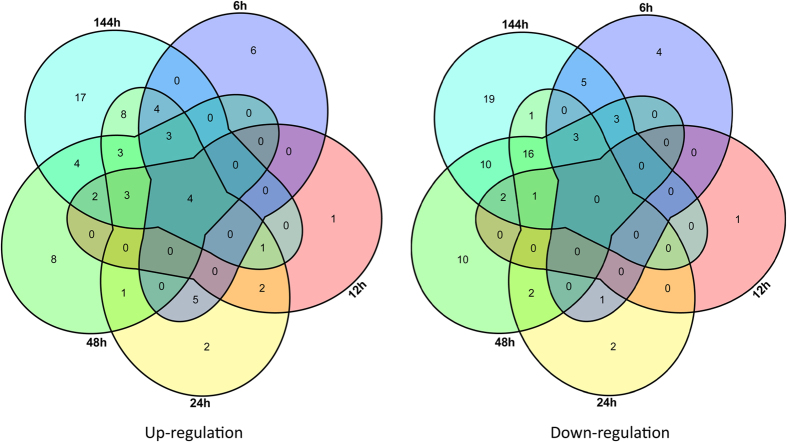
Venn diagram of differentially expressed proteins. The sum of the numbers in each large circle represents the total number of differentially expressed proteins among various combinations; the overlapping parts of the circles represents common differentially expressed proteins between combinations.

Among proteins that showed a significant change (*P* < 0.05) in abundance, 151 differentially expressed proteins (DEPs) were chosen by a ratio >1.50 or <0.67 at least in 1 time point under high temperature stress (Supplementary Information Table S2), representing 7.97% of the total identified proteins. In the control (0 h), and at 6 h, 12 h, 24 h, 48 h, and 144 h of high temperature stress, 22, 13, 36, 28, and 49 proteins were up-regulated, while 16, 4, 26, 47, and 60 proteins were down-regulated, respectively (Fig. 2). Among these proteins, the number (17) of DEPs was the least between 12 h and 0 h, and the number (109) of DEPs was the most between 144 h and 0 h. We then analyzed the Venn diagram of the DEPs at different time points (Fig. 3). This diagram shows that different proteins work at different time points. Additionally, 4 DEPs were up-regulated at all time points: 2 disulfide isomerases, 1 hsp70, and 1 unknown protein.

### Functional Classification of Differentially Expressed Proteins

**Figure 4:**
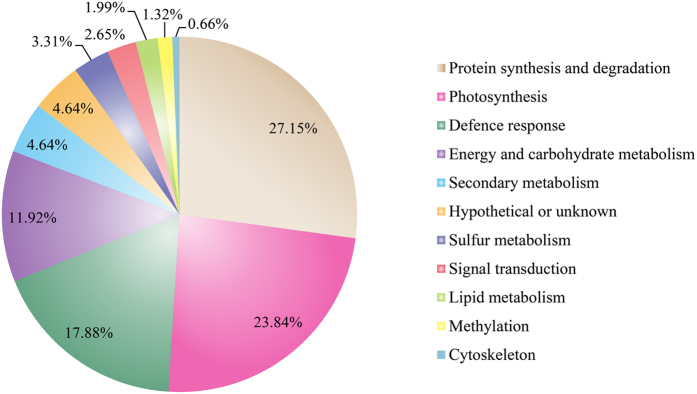
Functional categorization of the 154 differentially expressed proteins.

DEPs are classified into 11 categories according to their putative biological functions (Supplementary Information Table S3). Figure 4 shows these functional classification. The majority of DEPs (80.79%) are classified into 4 categories: protein synthesis and degradation (27.15%), photosynthesis (23.18%), defense response (17.88%), and energy and carbohydrate metabolism (12.58%). The other categories are as follows: secondary metabolism (4.64%), sulfur metabolism (3.31%), signal transduction (2.65%), lipid metabolism (1.99%), methylation (1.32%), cytoskeleton (0.66%), and hypothetical or unknown (4.64%).

**Figure 5:**
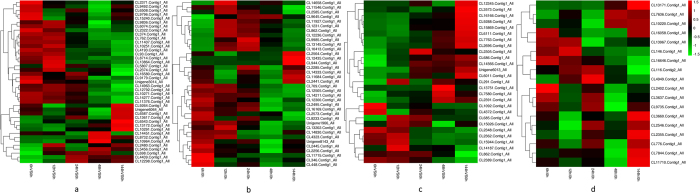
Hierarchical clustering of differentially expressed proteins with similar functions under high temperature stress. (**a**) Proteins related to protein synthesis and degradation; (**b**) Proteins related to photosynthesis; (**c**) Proteins related to the defense response; (**d**) Proteins related to energy and carbohydrate metabolism.

Based on hierarchical cluster analysis, we grouped the DEPs in the main categories during high temperature stress (Fig. 5). For protein synthesis and degradation (Fig. 5a), several enzymes involved in protein synthesis were down-regulated. On the contrary, proteins related to degradation were up-regulated. For photosynthesis (Fig. 5b), most proteins decreased, including photosystem II (PSII)-related proteins, phycobiliproteins, and proteins involved in the Calvin cycle. In contrast, for the defense response (Fig. 5c), many proteins were up-regulated, such as molecular chaperones, antioxidant enzymes, and metacaspases. Lastly, for energy and carbohydrate metabolism (Fig. 5d), several proteins participated in glycolysis and the citric acid cycle were up-regulated.

## Discussion

In recent years, transcriptomics and proteomics technologies have been widely applied to identify stress-responsive genes and proteins that are regulated by environmental stress in aquatic organisms^18^. Compared to transcriptomics, proteomics provides not only information at a mechanistic level but can also capture changes in protein activity measured as post-translational modifications^19^. Previously reported *P. haitanensis* proteomics studies were limited to 2D gel electrophoresis analysis^6^. However, this technique has several limitations, such as the low number of proteins it can identify and quantification inaccuracy, which limits its application for comprehensive analysis of proteome changes^20^. Although most proteomics studies in plants and algae have used 2D gel approaches, alternative methods such as iTRAQ and ICAT are now available^21^. In the present study, based on the iTRAQ technique, we successfully identified 151 proteins showing significant differential accumulation after high temperature stress. These proteins provide new insights into the mechanism of *Pyropia* responding to heat stress.

### Protein Synthesis and Degradation

High temperature stress can repress the synthesis of proteins^22^,^23^. In this study, several proteins involved in protein synthesis were down-regulated, including threonine and tryptophanyl-tRNA synthetase, ribosomal proteins, transaminases, threonine synthase, choline dehydrogenase, and nitrite reductase. However, it seems strange that some eukaryotic initiation factors (eIF) and protein synthesis elongation factors were up-regulated under high temperature stress (Supplementary Information Table S3). This may reveal the complexity of expression patterns for initiation and elongation factors following thermal stress^24^. In some previous studies, a high accumulation of eIF has been reported to induce cellular reorganization leading to PCD on a long-term exposure to high temperature stress^25^,^26^. Some other previous studies have also implicated the protein synthesis elongation factor in plant response to high temperature stress^27^,^28^. Elongation factors display chaperone activity, and it has been suggested that high temperature-induced accumulation of elongation factors is important for high temperature stress tolerance in plants. For example, cultivars expressing a higher elongation factor under high temperature stress were more tolerant to high temperature stress^29^.

High temperature stress is also associated with an enhanced risk of improper protein folding and denaturation^30^. The misfolded and denatured proteins may accumulate in cells under high temperature stress, which, if unchecked, leads to cell death. Two strategies solve this problem: refolding them or removing them. Several disulfide-isomerases, which play important roles in the folding of nascent polypeptides and in the proper formation of disulfide bonds in protein folding^31^, were observed to be up-regulated after heat treatment. Proteases, which remove the unfolded, denatured proteins and release amino acids for recycling^32^, were also up-regulated. Other proteins involved in removing the misfolded and denatured proteins were up-regulated as well, such as the glycine cleavage system p-protein and polyubiquitin (Supplementary Information Table S3).

Furthermore, vacuolar sorting protein, responsible for transport, was up-regulated under high temperature stress. This may provide cells with extra flexibility to cope with changing environmental conditions^33^. These results suggest when under high temperature stress, *P. haitanensis* reduced the production of proteins to avoid misfolding, and increased some enzymes to remove misfolded and denatured proteins.

### Photosynthesis

Due to its complex mechanisms and requirement for enzymes, photosynthesis is particularly sensitive to heat stress. Changes in environmental temperature are reflected by photosynthesis, which triggers a response aimed at achieving the best possible performance under new conditions^34^. In this study, we identified 35 DEPs involved in photosynthesis, which accounted for 23.18% of all DEPs.

The first step of photosynthesis is light absorption. In contrast to higher plants, *Pyropia* has intricate light-harvesting systems comprised of phycobilisomes (PBSs), which are made of phycobiliproteins (PBPs) and linker polypeptides^35^. PBPs are divided into 3 main groups: allophycocyanin (APC), phycocyanin (PC), and phycoerythrin (PE). Light energy absorbed by PE migrates first to PC, then to APC, and finally to chlorophyll a^36^. In our study, we found 2 APC and 1 PE (Supplementary Information Table S3). Down-regulated expression of these proteins under high temperature stress indicated that their capacity for transferring light energy decreased. It is interesting that a light harvesting protein sharply increased at 144 h of heat treatment while APC and PE decreased (Supplementary Information Table S3). This may suggest that the protein takes charge of absorbing light under long-term heat stress. Additionally, a linker polypeptide was detected to be up-regulated. It functioned like linker polypeptides, which stabilize the PBS structure and optimize absorbance characteristics and energy transfer^37^.

PSII is more thermolabile than photosystem I (PSI), and has been long believed to be a prominent heat sensitive component of photosynthesis as its activity can be greatly reduced or even partially stopped under high temperature stress^38^. In the present study, the abundance of many proteins related to PSII was down-regulated, such as PsbU, PsbB, PsbS, PsbQ, cytochrome c550, and low PSII accumulation protein. However, PsaA, which is involved in PSI, was up-regulated (Supplementary Information Table S3). This means that high temperature stress can significantly decrease the PSII activity in *P. haitanensis*, but not in PSI, and the PSI-driven cyclic electron flow allows *Pyropia* to survive in stress conditions^39^,^40^.

Fructose-1,6-bisphosphatase (FBPase), sedoheptulose-1,7-bisphosphatase (SBPase), fructose bisphosphate aldolase, ribose-5-phosphate isomerase, and transketolase, which are involved in the Calvin cycle^41^, decreased under high temperature stress. Moreover, ferredoxin also decreased. This protein can modulate the activities of FBPase and SBPase^42^. The down-regulated expression of these proteins further suggests that high temperature stress inhibited photosynthesis in *P. haitanensis*.

Although most photosynthesis-related proteins down-regulated under heat stress, it is worth mentioning that carbonic anhydrase (CA) and oxygen-evolving enhancer protein 1 (OEE) were up-regulated (Supplementary Information Table S2). CA plays a crucial role in the CO_2_-concentrating mechanism (CCM) in algae^43^. In a previous study, several CA genes (*PhαCA1, PhβCA2, PhβCA3*, and *PhγCA1*) in *P. haitanensis* were found up-regulated during high temperature stress^44^. High temperature stress inhibits the utilization of inorganic carbon in *P. haitanensis*, and the higher the temperature, the lower the utilization^45^. As the key enzyme of inorganic carbon utilization, the CA level must be up-regulated to maintain the equilibrium between CO_2_ and HCO_3_^−^ during stress. In addition to function in CCM, CA also shows antioxidant activity and plays a role in the hypersensitive defense response^46^. Expression of a rice carbonic anhydrase gene (*OsCA1*) and CA activity were up-regulated by some environmental stresses, and a transgenic *Arabidopsis* overexpressing *OsCA1* had a greater salt tolerance at the seedling stage than wild-type plants^47^. Additionally, recent research in *Capsosiphon fulvescens* showed that OEE is also an excellent antioxidant^48^.

Based on the expression of proteins related to photosynthesis, we conclude that *P. haitanensis* limited damage to a repairable level by reducing photosynthesis under high temperature stress.

### Defense Response

Twenty-seven identified DEPs were functionally characterized as being involved in the defense response (Supplementary Information Table S3), which was classified into molecular chaperone, redox homeostasis, programmed cell death, and others.

An essential component of acquired thermotolerance in plants and other organisms is the induction and synthesis of molecular chaperones or heat shock proteins (HSPs)^49^. A wide range of proteins have chaperone activity. Moreover, many molecular chaperones were originally identified as heat-shock proteins^50^. In plants, well-characterized HSPs are grouped into 5 different families: HSP100, HSP90, HSP70, HSP60, and sHSP^51^. Excluding HSP100, all other heat-shock proteins identified in the present study were up-regulated (Supplementary Information Table S3). Furthermore, HSP70 was the most abundant, indicating its fundamental role in response to high temperature stress^52^. The up-regulation of these HSPs prevent denaturation of other proteins caused by high temperature, and thus improve the high temperature tolerance of *P. haitanensis*. Calnexin is an endoplasmic reticulum-localized molecular chaperone protein involved in the folding and quality control of proteins^53^. Previous study identified that the accumulation of calnexin helps plants deal with abiotic stresses^54^. The up-regulation of calnexin observed in this study (Supplementary Information Table S3) may suggest that calnexin work as a molecular chaperone and play an important role in responding to high temperature stress in *P. haitanensis*.

High temperature stress may disturb cellular redox homeostasis and promote the production of reactive oxygen species (ROS). Excessive ROS could damage cellular components. To cope with oxidative stress, cells have developed a wide range of antioxidant systems^55^,^56^,^57^,^58^. In this study, several antioxidant enzymes were identified (Supplementary Information Table S3), including ascorbate peroxidase (APX), catalase (CAT), and methionine sulfoxide reductase (MSR). In contrast with the down-regulation of APX, CAT and MSR were up-regulated under high temperature stress. As an antioxidant, thioredoxin (TRX) can also protect cells from oxidative damage^59^. Although we could not find TRX in the DEPs, we did detect 2 related enzymes: methionine-S-oxide reductase, which is responsible for the TRX synthesis and was up-regulated under heat stress, and TRX reductase, which reduces TRX and was down-regulated. Furthermore, quinone oxidoreductase, which works as a superoxide scavenger^60^, increased under high temperature stress. The expression of these antioxidant enzymes suggest that they may play important roles in the pathway for scavenging ROS and contribute to the survival of *P. haitanensis* blades when under high temperature stress.

Programmed cell death (PCD) in plants is a crucial component of development and defense mechanisms^61^. PCD is triggered by the sequential activation of cysteine proteases known as caspases, and the inactive precursors of caspases (procaspases) is induced by the release of electron carrier protein cytochrome c^62^. Supplementary Information Table S2 shows that cytochrome c maintained its expression before 48 hours of heat treatment. We also observed an increase in metacaspase. This may suggest that PCD is also involved in the high temperature response of *P. haitanensis*. Accumulating evidence shows that PCD is triggered by different environmental factors such as high light, ultraviolet radiation, drought, salt, heat, cold, or flooding. Upon reaching certain thresholds with these changes, plant cells can no longer maintain proper metabolic processes and PCD is induced^63^. Although it is unfavorable for biomass production, the selective death of cells and tissues under abiotic stresses eventually provides survival advantages for the whole organism^63^.

Other stress-related proteins such as polyamine oxidase, stress-induced protein, and aldehyde dehydrogenase, were up-regulated to deal with high temperature stress (Supplementary Information Table S3). Several reports have shown that these proteins play a part in abiotic stress responses in plants^64^,^65^.

In conclusion, the defense response of *P. haitanensis* under high temperature stress is complicated and involves molecular chaperones, antioxidant systems, PCD, and other stress-related proteins. These proteins work together to maintain cellular and redox homeostasis.

### Energy and Carbohydrate Metabolisms

Plants respond to stress conditions by triggering a network of events linked to energy and carbohydrate metabolism^66^. As expected, energy and carbohydrate metabolism were affected by heat in this research. The DEPs involved in energy and carbohydrate metabolism were divided into 4 groups: glycolysis, citric acid cycle, pentose phosphate pathway, and others (Supplementary Information Table S3).

Glycolysis is a metabolic pathway that oxidizes glucose to generate ATP, reductant, and pyruvate^67^. Under high temperature, all glycolysis-related proteins were up-regulated, including glucose-6-phosphate isomerase, enolase, transaldolase, and glyceraldehyde 3-phosphate dehydrogenase. The citric acid cycle is a series of chemical reactions that produce energy. Citrate synthase and aconitate hydratase, which belong to this pathway, were up-regulated under heat stress. Similar to glycolysis, the pentose phosphate pathway generates NADPH and pentoses. Apart from the above-mentioned enzymes, glucose-6-phosphate 1-dehydrogenase and ribulose-phosphate 3-epimerase were inhibited by heat stress. Furthermore, other proteins such as galactose kinase, UDP-glucose 4-epimerase, V-type proton ATPase, and ATP/ADP translocator, were up-regulated. These proteins are core members in energy and carbohydrate metabolism. In our study, due to many proteins being involved in the complex network of energy metabolism, it is unsurprising that proteins with different abundance profiles were identified. These results suggest that the blades of *P. haitanensis* require high energy levels to fuel defense mechanisms and repair damage under high temperature stress.

### Secondary Metabolism

Secondary metabolites of plants often refer to compounds that have no fundamental role in the maintenance of life processes but are important for interaction with the environment for adaptation and defense^68^. Several enzymes that participate in isoprenoid, terpenoid, riboflavin, and nucleotide sugars metabolism decreased in the present research. They are 4-hydroxy-3-methylbut-2-en-1-yl diphosphate synthase, farnesyltransferase, 1-deoxy-D-xylulose 5-phosphate reductoisomerase, FAD synthetase, and UDP-sulfoquinovose synthase. A previous study showed that stressed plants are expected to have reduced metabolic activities because of a reduced level of photosynthetic activity and reduced uptake of nitrogen from the soil^69^. Our results confirmed this point.

### Sulfur Metabolism

Sulfur is a ubiquitous and essential element for plants^70^. Sulfur metabolism is important for biochemical and physiological processes, including redox cycles, enzyme activity regulation, and heavy metal and xenobiotic detoxification. In plants, abiotic stress affects sulfur metabolism, leading to the activation of a wide range of adaptive responses^71^,^72^. Five dysregulated proteins were classified into sulfur metabolism (Supplementary Information Table S3), while cysteine synthase and sulfate adenylyltransferase are enzymes involved in sulfur assimilation. The up-regulation of these proteins suggests that sulfur is required for *P. haitanensis* to adapt to heat stress.

### Signal Transduction

The process by which plant cells sense stress signals and transmit them to cellular machinery to activate adaptive responses is referred to as signal transduction^73^. In other words, the initial high temperature stress signals must be transmitted by some signaling molecules and trigger downstream signaling processes and transcription controls, which in-turn activate stress-responsive mechanisms to reestablish homeostasis and protect and repair damaged proteins and membranes. In the present study, the receptor of activated protein kinase C (RACK) was up-regulated. RACK regulates various signaling pathways ranging from developmental processes to immune and stress responses in plants^74^. Additionally, 2 proteins that participate in inositol metabolism were found. Inositol serves as an important component of the phosphoinositide pathway, which is essential for plant survival in a changing environment^75^,^76^. The up-regulation of these 2 proteins indicate that the phosphoinositide pathway may play important roles in the signal transduction of *P. haitanensis* when under high temperature stress.

### Lipid Metabolism

Lipids are linked to many cellular functions including storage for energy generation and membrane synthesis^77^. Previous research found that under high temperature stress, *P. haitanensis* increased energy metabolism to maintain physiological balance^7^. In this study, acyl-CoA binding protein and glyoxysomal beta-ketoacyl-thiolase, which participates in fatty acid beta-oxidation, increased at high temperature. This expression reflects that when coping with high temperature stress, *P. haitanensis* can also use lipids as an energy source.

### Methylation

DNA methylation is an important mode of epigenetic modification in plants. It plays vital roles in regulating growth and development in plants, and is involved in responses to a variety of stresses. Environmental stimuli adjust gene networks associated with the stress response by changing the level and pattern of DNA methylation, thereby enhancing adaptation to various stresses^78^. S-adenosyl-methionine (SAM)-dependent methyltransferase, which utilizes the methyl donor (SAM) as a cofactor to methylate nucleic acids, was down-regulated. This may increase the expression of some genes and proteins, thus protecting *P. haitanensis* from abiotic stress.

### Cytoskeleton

The cytoskeleton in plant cells plays important roles in controlling cell shape and mediating intracellular signaling, and can undergo profound changes when under abiotic stress^79^. Tubulin is the major constituent of microtubules. It binds 2 moles of GTP, one at an exchangeable site on the beta chain and one at a non-exchangeable site on the alpha chain. Here, the up-regulation of the tubulin beta chain in response to high temperature stress indicated its function in *P. haitanensis* cellular homeostasis.

## Conclusions

**Figure 6:**
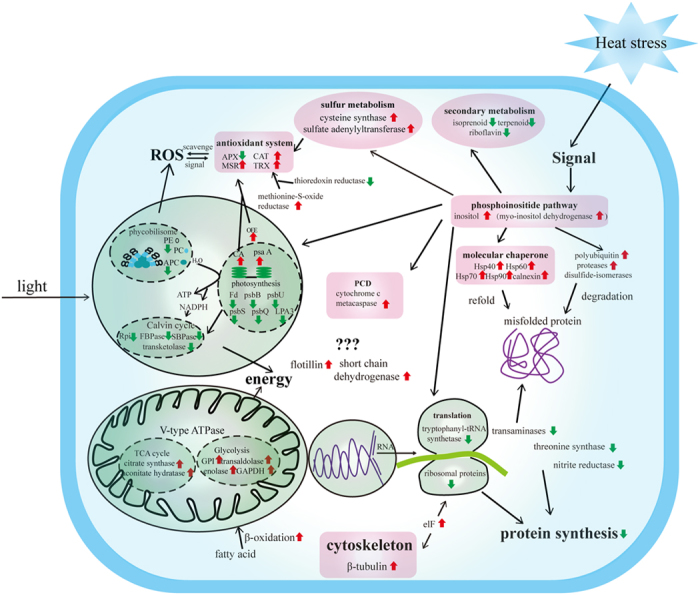
Cell diagram of *Pyropia haitanensis* mechanisms involved in high temperature stress tolerance. Down-regulated proteins are indicated by 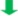, whereas up-regulated proteins are indicated by 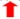, hypothetical or unknown proteins are indicated by ???.

A significant number of stress-responsive proteins were identified from the Z-61 stain of *P. haitanensis* using the iTRAQ technique. The expression of these proteins shows that there is a clear response to high temperature in Z-61 (Fig. 6). The mechanism is as follows:

Under high temperature stress, photosynthesis, protein synthesis, and secondary metabolism are inhibited to limit damage to a repairable level. As time progresses, misfolded proteins and ROS accumulate, which are harmful to cells. This leads to the up-regulation of molecular chaperones, proteases, and antioxidant systems. It is inevitable that some cells are injured by high temperature, but PCD aims to remove them. Furthermore, sulfur assimilation and the cytoskeleton efficiently maintain cellular and redox homeostasis. In this process, the phosphoinositide pathway works as the major player for signal transduction. All these aforementioned behaviors require significant energy. Therefore, glycolysis, the citric acid cycle, and beta-oxidation of fatty acids are up-regulated. In summary, all observed changes were aimed at establishing a new steady-state balance of metabolic processes to enable continual Z-61 function and survival under higher temperatures.

## Materials and Methods

### Plants and Stress Treatment

The Z-61 strain of *P. haitanensis*, which is tolerant to high temperatures and grows with a high yield, were used in this study. Samples were selected and purified by researchers working in the Laboratory of Germplasm Improvements and Applications of *Pyropia* in Jimei University, Fujian Province, China. Sixty 15 ± 2 cm long blades of Z-61 were randomly selected and cultured in 12 aerated flasks (2000 mL) with 5 blades each at 29 °C (high temperature), and were illuminated by 50–60 mol m^−2^s^−1^ photons (10:14, L:D) for 0, 6, 12, 24, 48, and 144 hours. Each sample had 2 biological replicates. The cultured medium was natural seawater enriched with MES medium.

### Protein Extraction

Blade proteins were extracted according to a previously published method^80^. Briefly, blades were ground in a precooled mortar in the presence of liquid nitrogen. Approximately 0.2 g of the homogenate was precipitated for 2 h with 10 mL acetone, 10% trichloroacetic acid, 0.07% β-mercaptoethanol (BME), and 0.2% polyvinylpyrrolidone at −20 °C. After centrifugation at 15,000 × g for 20 min at 4 °C, the supernatant was removed and the pellet was rinsed 3 times in ice-cold acetone (including 0.07% BME). Between rinsing steps, samples were incubated for 30 min at 25 °C. Then, the pellet was air-dried, resuspended in 5 mL lysis buffer (7 M urea, 2 M thiourea, 40 mM Tris-HCl, 65 mM dithiothreitol (DTT), 4% CHAPS, 1 mM PMSF, and 2 mM EDTA), and vortexed for 1 h at room temperature. The homogenate was centrifuged at 15,000 × g for 20 min at 4 °C, and the supernatant was collected and mixed well with ice-cold acetone (1: 4, v/v) with 30 mM DTT. After repeating this step twice, supernatants were collected and stored at −80 °C for iTRAQ analysis.

### Protein Digestion and iTRAQ Labeling

For digestion, the 300-μg protein pellet from the previous step was resuspended in digestion buffer (100 mM triethylammonium bicarbonate (TEAB), 0.05% w/v sodium dodecyl sulfate (SDS)) to a final concentration of 1 mg/mL (total protein measured by bicinchoninic acid assay (Sigma, St. Louis, MO)). Equal aliquots (500 μg) from each lysate were then digested with trypsin (Sigma; 1: 40 w/w) overnight at 37 °C in a sealed tube. The tryptic peptides were then lyophilized and dissolved in the 50% TEAB buffer, and the trypsin digested samples were analyzed using MALDI-TOF/TOF to ensure complete digestion.

The iTRAQ labeling of peptide samples derived from each time point of high temperature stress was performed using iTRAQ Reagent-8plex Multiplex Kit (Applied Biosystems, Foster City, CA) according to the manufacturer’s protocol. For each time point (i.e., 0 h, 6 h, 12 h, 24 h, 48 h, or 144 h), 12 samples (2 biological replicates) were iTRAQ labeled. Peptides were labeled with respective isobaric tags (113 for 0 h; 114 for 6 h; 115 for 12 h; 116 for 24 h; 117 for 48 h; and 118 for 144 h), incubated for 2 h, and vacuum centrifuged to dryness.

Labeled peptides were then fractionated using Polysulfoethyl A SCX columns (100 × 4.6 mm, 5 μm particle size, 200A° pore size) using an HPLC system (Shimadzu, Japan) at a flow rate of 1.0 mL min^−1^. Retained peptides were eluted using Buffer A (10 mM KH_2_PO_4_ in an aqueous solution of 25% acetonitrile acidified to a pH of 3.0 with H_3_PO_4_) and Buffer B (Buffer A with 500 mM KCl). The following chromatographic gradients were applied: 0–25 min 100% Buffer A; 25–32 min 90% Buffer A and 10% Buffer B; 32–42 min 80% Buffer A and 20% Buffer B; 42–47 min 55% Buffer A and 45% Buffer B; 47–60 min 100% Buffer B; 60–75 min 100% Buffer A. Chromatograms were recorded at 214 nm. The collected fractions were desalted with Sep-Pak Vac C18 cartridges (Waters, Milford, Massachusetts), concentrated to dryness using vacuum centrifuge, and reconstituted in 0.1% formic acid for LC-MS/MS analysis.

Protein digestion and iTRAQ labeling was conducted in 2 technical replicates.

### LC-MS/MS Proteomic Analysis

The SCX peptide fractions were pooled to obtain 10 final fractions and reduce the number of samples and collection time. A 10-μL sample from each fraction was injected 3 times to the Proxeon Easy Nano-LC system. Peptides were separated on a C18 analytical reverse phase column (ID 75 μm × 10 cm, 200Å, 3 μm particles) at a flow rate of 250 nL/min and a linear LC gradient profile was used to elute peptides from the column. We then analyzed the fractions using a Q Exactive mass spectrometer (Thermo Fisher Scientific, MA, USA). Data were acquired using a data-dependent acquisition mode in which, for each cycle, the 10 most abundant multiply-charged peptides (2+ to 4+) with an m/z between 300 and 1800 were selected for MS/MS with 15 s dynamic exclusion setting. The fragment intensity multiplier was set to 20 and the maximum accumulation time was 2 s. Peak areas of the iTRAQ reporter ions reflected the relative abundance of proteins in the samples.

### Data Analysis

For iTRAQ/Shotgun protein identification, the raw mass data were processed with Proteome Discover 1.3 (Thermo Fisher Scientific) and searched with in-house MASCOT software 2.3.02 (Matrix Science, London, U.K.) against the *P. haitanensis* UniGene database, which was obtained from whole transcriptome sequencing^12^. In the database search, all parameters were set as follows: specifying trypsin as the digestion enzyme; the carbamidomethylation of cysteine and iTRAQ 8plex modification of the N terminus and K as fixed modifications; iTRAQ 8plex modification of Y and oxidation on methionine as variable modification; the initial precursor mass tolerance to 20 ppm and the fragment ion level to 0.1 Da; and the maximum missed cleavages as 2. The data files generated by MASCOT were processed using Scaffold Q+ (Proteome Software, Portland). For iTRAQ quantification, the peptide for quantification was automatically selected by the algorithm to calculate the reporter peak area, error factor (EF), and *p* value. The resulting data set was auto bias-corrected to remove any variations due to unequal mixing during combining differently labeled samples. The average value of 2 biological replicates and 2 technical replicates were used to indicate the final protein abundances at a given time point. Proteins with a 1.5-fold change between samples and a *p* value less than 0.05 were determined as differentially expressed proteins.

### Principal Component Analysis and Venn Diagram Analysis

Principal component analysis (PCA) analysis was performed using the prcomp package, calculation was based on a singular value decomposition, and Fig. 1 were drawn by OriginPro 9.1. Venn diagram analysis was performed online (http://www.omicshare.com/tools/index.php/Home/Soft/venn).

### Functional Classification and Enrichment Analysis

Differently expressed proteins were functionally classified according to MapMan ontology^81^. Enrichment analysis was conducted using the singular enrichment analysis (SEA) tool in the agriGO toolkit^82^. Metabolic pathway enrichment analysis of responsive proteins was conducted according to information from the KEGG Pathway Database.

## Additional Information

**How to cite this article:** Shi, J. *et al*. Differential Proteomic Analysis by iTRAQ Reveals the Mechanism of *Pyropia haitanensis* Responding to High Temperature Stress. *Sci. Rep.* **7**, 44734; doi: 10.1038/srep44734 (2017).

**Publisher's note:** Springer Nature remains neutral with regard to jurisdictional claims in published maps and institutional affiliations.

## Supplementary Material

Supplementary Information

Supplementary Dataset
