# Supplementary material for: Differential Proteomic Analysis by iTRAQ Reveals the Mechanism of Pyropia haitanensis Responding to High Temperature Stress
Source: Sci Rep. 2017 Mar 17;7:44734. doi: 10.1038/srep44734 (PMC5356179; doi:10.1038/srep44734)
Supplement: Supplementary Information [file srep44734-s1.doc]

**Supplementary Information**

**Differential proteomic analysis by iTRAQ reveals the mechanism of *Pyropia haitanensis* responding to high temperature stress**

Jianzhi Shi^1,2^, Yuting Chen^1,2^, Yan Xu^1,2^, Dehua Ji^1,2^, Changsheng Chen^1,2^, Chaotian Xie^1,2 *^

Table S1. Overview of iTRAQ experiment.

Table S2. Identified proteins in the iTRAQ experiment.

Table S3. Differentially expressed proteins and their relative expression levels under high temperature stress.
